# Psychosocial Correlates of Dietary Behaviour in Type 2 Diabetic Women, Using a Behaviour Change Theory

**Published:** 2014-06

**Authors:** A. Didarloo, D. Shojaeizadeh, R. Gharaaghaji asl, S. Niknami, A. Khorami

**Affiliations:** ^1^Social Determinants of Health Research Center, Department of Health and Community Medicine, School of Medicine, Urmia University of Medical Sciences, Urmia, Iran; ^2^Department of Health Education and Promotion, School of Public Health, Tehran University of Medical Sciences, Tehran, Iran; ^3^Department of Biostatistics and Epidemiology, School of Medicine, Urmia University of Medical Sciences, Urmia, Iran; ^4^Department of Health Education and Promotion, School of Medical Sciences, Tarbiat Modares University, Tehran, Iran; ^5^Department of Nursing and Midwifery, Urmia University of Medical Sciences, School of Nursing and Midwifery, Urmia, Iran

**Keywords:** Diabetic women, Dietary behaviour, Self-efficacy, Theory of Reasoned Action, Iran

## Abstract

The study evaluated the efficacy of the Theory of Reasoned Action (TRA), along with self-efficacy to predict dietary behaviour in a group of Iranian women with type 2 diabetes. A sample of 352 diabetic women referred to Khoy Diabetes Clinic, Iran, were selected and given a self-administered survey to assess eating behaviour, using the extended TRA constructs. Bivariate correlations and Enter regression analyses of the extended TRA model were performed with SPSS software. Overall, the proposed model explained 31.6% of variance of behavioural intention and 21.5% of variance of dietary behaviour. Among the model constructs, self-efficacy was the strongest predictor of intentions and dietary practice. In addition to the model variables, visit intervals of patients and source of obtaining information about diabetes from sociodemographic factors were also associated with dietary behaviours of the diabetics. This research has highlighted the relative importance of the extended TRA constructs upon behavioural intention and subsequent behaviour. Therefore, use of the present research model in designing educational interventions to increase adherence to dietary behaviours among diabetic patients was recommended and emphasized.

## INTRODUCTION

Type 2 diabetes mellitus (T2DM) is a major global public-health challenge faced in the 21st Century (1). T2DM is the fourth or fifth leading cause of death in most developed countries, and there is a growing evidence that it has reached epidemic proportions in many developing and newly-industrialized countries (2).

Most studies have confirmed that changes in lifetyle can prevent its incidence, and proper control of blood glucose can reduce the risk of complications (3-5). Dietary management among patients with T2DM is one way to prevent or delay the long-term effect of the condition. Diabetic people are routinely advised to adopt a healthful diet; dietary changes include modifications in food habits and meal patterns on a lifelong basis (6,7). However, diet-related lifestyle behaviour is associated with very low compliance among the diabetics (8,9).

To develop effective dietary interventions for diabetic patients, it is necessary to understand the factors that determine eating behaviour in these populations. An understanding of such determinants enables the planning of health promotion interventions targeted at changing behaviour. Lifestyle behavioural change interventions are likely to be more successful when these focus on theory-based determinants (10).

Theory-based research is fundamental to the understanding of health behaviours by providing a framework by which to examine the relationships among constructs, to assess the impact of the various constructs, and to delineate factors and determinants to be studied (11-13).

There are several behaviour change models/theories. One is Aizen's Theory of Planned Behaviour (TPB). Since the earliest day of TPB, there has been a degree of uncertainty concerning between Aizen's perceived behavioural controls (PBC) and Bandura's self-efficacy (SE) construct. Both constructs concern the execution of a behaviour (14,15). On the other hand, Giles *et al*. (16) suggested that the predictive utility of the TPB may be enhanced by replacing PBC with self-efficacy.

Based on the above documents, we utilized Theory of Reasoned Action, along with self-efficacy (TRA+SE) to predict factors influencing dietary practice among type 2 diabetic women. This theoretical framework has proved its efficacy in predicting several health-related behaviours, including weight loss, smoking, alcohol-abuse, HIV risk behaviours, and mammography screening.

The Theory of Reasoned Action (TRA) is a widely-used behavioural prediction theory which represents a social-psychological approach to understanding and predicting the determinants of health-related behaviour (17,18). The TRA+SE model suggests that intention is directly driven by three major constructs: attitude, subjective norm, and self-efficacy; the stronger the intention, the more likely an individual will perform the behaviour (19). Attitude is known as the degree to which an individual has a favourable or unfavourable evaluation of the behaviour; subjective norm measures the importance others hold about performing or not performing a behaviour and one's willingness to comply with those referents; self-efficacy refers to an individual's confidence in his or her ability to perform a behaviour in various situations. Self-efficacy has been recognized as an important mediating variable between knowledge, attitudes, skills, and behaviour (20).

Self-efficacy is the focal belief on which human motivation and action is founded: unless a person believes he/she can produce desired effects by his/her own actions, he/she has little incentive to act or to persevere in the face of difficulties. Highly-efficacious individuals tend to tackle more challenging tasks, employ better strategies, put forth more sustained effort, and be more persistent in the face of obstacles, setbacks, and difficulties (15,21,22). A self-efficacious individual persists because he/she believes he/she will eventually succeed. High self-efficacy minimizes stress and, hence, improves performance (23). As in the original formulations of the social cognitive theory by Albert Bandura, self-efficacy might not only influence one's intention to act but also has a direct effect on behaviour (22). The authors decided to test this theoretical framework on dietary practice among diabetic women. It seems that results of this project can add new information to previous documents and can guide researchers, general practitioners, and educators regarding preventing and controlling diabetes.

## MATERIALS AND METHODS

This research is a cross-sectional survey to examine factors influencing dietary behaviour among Iranian women with type 2 diabetes. A convenient sample, including 352 women with type 2 diabetes referred to the Khoy Diabetes Clinic, Iran, was recruited for the present study.

To collect data, we utilized a self-administered questionnaire. This measurement instrument included 48 items that measure sociodemographic characteristics, knowledge of diabetic patients, and variables of the proposed model.

Response categories for each item relating to the proposed model variables include a 5-point Likert scale ranging from 1 (strongly disagree) to 5 (strongly agree) and a 5-point Likert scale ranging from 1 (highly unimportant) to 5 (highly important).

Participants's attitude and subjective norm towards dietary behaviour were indirectly measured by separate scales.

Self-efficacy towards dietary practice was assessed using statements, such as “I am able to regulate my blood sugar level by eating healthy foods” and “I think I'm able to control dietary regimen when I am in train or party”, etc.

Behavioural intention was measured with the statements: “I intend to control my dietary programme regularly within the coming month” and “I will plan to consider dietary recommendations if my physician prescribes for me.”

To assess the level of healthy eating performance, we used subscale of dietary behaviour from the Summary of Diabetes Self-care Activities Measure (24). Knowledge of diabetes was assessed by an 11-item scale. Each correct response scored 2, and each ‘don't know’ response scored 0, with the response category ranging from 0 to 22. The subsequent results were based on the mean knowledge derived from 11 questions, each with a maximum point of 2 and a minimum point of 0.

To test content validity, the self-administered questionnaire was shown to a panel of experts. They evaluated each item for its distinctiveness, understandability, and appropriateness for the purpose of the study, and final revisions were made based on their comments.

To evaluate reliability, instruments were completed by 352 diabetic patients, and Cronbach's alpha for each questionnaire was calculated. Internal consistency of the knowledge questionnaire was assessed, and its Cronbach's alpha was 0.77. Similarly, internal consistency of other sets of questionnaire was studied, and Cronbach's alpha values for attitude, subjective norms, self-efficacy, behavioural intention, and dietary behaviour in the questionnaire were computed and confirmed (0.75, 0.73, 0.80, 0.72, and 0.78 respectively).

After assessing the psychometric characteristics of the instruments, the sets of questionnaire were completed by study subjects. Statistical analyses in SPSS software (version 16.0) were performed using descriptive and inferential statistical methods. In this study, a p value of less than 0.05 was considered significant.

## RESULTS

Approximately 50.9% of the subjects were between 44 and 56 years of age. Slightly more than half of the research subjects (53%) were non-literate, 93.2% had no formal jobs, except house-keeping, and the remaining (6.8%) had part-time jobs. About 91% of studied samples were currently married, and nearly half of the women had a moderate monthly income. Most diabetic women were obese (40.1%) and overweight (46.9%). Most of the participants (62.2%) had diabetes lower than 10 years. Approximately three-fourths of the subjects (74.4%) were being treated by oral hypoglycaemia agents; 22.2% of cases were using other therapeutic methods; the remaining participants (3.4%) were not receiving any treatment. About two-thirds of the samples (63.1%) were visited by their physicians every 1 to 2 month(s) and the rest less frequently. Based on findings of the study, about 78.1% of the participants had not previously participated in any formal instructional meetings. The majority of subjects (72.4%) reported that they acquired diabetes-related information from health professionals.

Inferential statistical methods were also used for analyzing data of the current study. Correlation matrix coefficients were applied to reveal the association of the used model constructs together, especially between independent and dependent variables. These are presented in Table 1. To determine the actual determinants of intention and dietary practice in participants, we utilized two models of Enter multiple linear regressions.

In the first model, sociodemographic variables were entered in regression equation to highlight effective factors on intention and behaviour of the diabetics. In the final model, both significant sociodemographics in the first model and the model constructs were placed in regression equation. The most important point in this study is that a factor is considered actual predictor when it is significant in the final model of regression analysis.

The only construct that was shown to be predictor of behavioural intention and dietary behaviour was self-efficacy. However, except self-efficacy and intention, other constructs of the proposed model had no direct correlation with behaviour. Among sociodemographics, there is no variable that predict intent to perform dietary behaviour but visit interval and source of obtaining information predicted dietary practices as presented in Table 2 and 3. Findings showed that the theoretical framework used in this study explained 31.6% of variance of behavioural intention and 21.5% of variance of dietary behaviour.

**Table 1.** Correlations among the extended TRA model constructs (N=352)

Construct	1	2	3	4	5
Dietary behaviour	1				
Intention	0.323**	1			
Attitude	0.253**	0.412**	1		
Subjective norm	0.213**	0.335**	0.294**	1	
Self-efficacy	0.375**	0.500**	0.461**	0.426**	1

**Correlation is significant at p=0.01 level (2-tailed)

**Table 2.** Enter multiple linear regression analyses of behavioural intention (N=352)

Parameter	Standardized coefficient (beta)	T value	p value
Model 1			
Sociodemographics			
Age	-0.071	-1.292	0.197
Education	0.091	1.481	0.139
Job	−0.039	−0.726	0.469
Marital status	0.121	2.306	0.022*
Income	0.099	1.845	0.066
Duration of disease	−0.096	−1.805	0.072
Treatment type	0.054	1.018	0.309
Visit interval	−0.133	−2.498	0.013*
Participation in educational sessions	−0.061	−1.155	0.249
Source of obtaining information	0.072	1.370	0.172
BMI	−0.018	−0.337	0.736
Knowledge of patients	0.080	1.353	0.177
Model 2			
Sociodemographics and the model constructs			
Marital	0.053	1.177	0.240
Visit interval	−0.081	−1.823	0.069
Subjective norm	0.128	2.572	0.011*
Self-efficacy	0.340	6.353	0.000**
Attitude	0.211	4.157	0.000**

*p<0.05;

**p<0.01

## DISCUSSION

Most studies have shown that physical activity and diet reduce the risk of developing diabetes. This investigation sought to identify factors influencing intention and dietary behaviour in a sample of type 2 diabetic women in Iran. It was found that intent and dietary behaviour of the diabetics can be affected by sociodemographics as external factors and constructs of the proposed model as internal elements. The prediction of each of these factors to intention and behaviour varied (Table 2 and 3). The results indicated that self-efficacy was the most powerful determinant of intention to perform dietary behaviour and indirectly affected behaviour (β=0.34, p<0.01). This has also been found in other TPB studies of dietary behaviours (25,26).

One study using constructs of the different theories showed that the effect of self-efficacy on a given behaviour through intention is stronger than other psychological and social factors, and this is consistent with our results (27). The stronger the diabetic patients’ self-efficacy beliefs are, the more reliably they will perform health behaviours (15). These results confirm the influence of self-efficacy in theory and theory-driven interventions. Therefore, if self-efficacy is used in extending theories and designing interventions, this will increase the ability of these programmes to change behaviour. To improve diet of the diabetics, healthcare professionals, especially dietitians and diabetes educators, could direct interventions on promoting diabetic patients’ self-efficacy beliefs towards eating a healthful diet. When patients feel a high self-efficacy, they will consider dietary regimens and select appropriate and healthy dietary choices.

In the current study, regression analysis results demonstrated that a relatively positive attitude towards dietary practice among the diabetics was the factor that most encouraged patients to adopt this behaviour (β=0.21, p<0.05). When people feel that a behaviour may lead to positive health outcomes, it is more likely to be adopted and maintained. This finding was supported by the results of previous research regarding the effects of attitudes on health-realted behaviours. For instance, Brekke *et al*. (28) and Nejad *et al*. (29) demonstrated that attitude was related to intention to eat a healthful diet.

Also, the results showed that social pressure/subjective norm is another theoretical framework construct that had significant positive correlation with dietary behaviour through intentions (β=0.13, p<0.05). Findings from other studies [like the one by Nguyen *et al*. (30)] also confirmed subjective norm to be associated with intent to eat a low-fat diet. Thus, this result was concordant with our finding regarding subjective norm. In other studies, subjective norm has not been found to be a significant variable (26,31). These findings were contrary to the results of this aspect of our study. These differences in findings are consistent with Ajzen and Fishbein's (17) view that people's beliefs about behaviours and the importance they place on these beliefs differ. Thus, for persons with diabetes, social influence may be an important determinant in dietary practices.

Based on the results of this study, self-efficacy predicted dietary behaviours more than behavioural intention/motivation. This finding was one of the most important points that have been observed in the present research. Results of regression analyses proved the above finding (Table 3). The findings indicated that self-efficacy was the strongest predictor both for behavioural intention and dietary behaviours. Therefore, it should be considered in designing educational interventions to increase adherence to dietary recommendations amongst diabetics.

In addition to the desired model variables, experiences and personal characteristics as external factors of the proposed model can also affect adherence to suitable dietary behaviours in diabetic women and can both directly and indirectly explain and predict them. In this study, among the characteristics, only visit interval of patients and source of acquiring knowledge about diabetes had significant association with dietary practices (p<0.01). When time interval of visiting patients reduces, the adoption of dietary recommendations about diabetes probably increases and vice-versa. Visiting the diabetic patients transforms a communication process between the patient and care provider; under this communication, patients’ knowledge regarding diabetes and management strategies and acquiring positive attitudes and self-efficacy would increase. In turn, intent to engage in behaviour and performing self-care practices would increase. This finding of the current study added support to the growing evidence showing the importance of provider-patient communication for promoting self-care behaviours in diabetes (32).

**Table 3.** Enter multiple linear regression analyses of dietary behaviour (N=352)

Parameter	Standardized coefficient (beta)	T value	p value
Model 1			
Sociodemographics			
Age	−0.048	−0.879	0.380
Education	0.053	0.870	0.385
Job	0.002	0.038	0.970
Marital status	0.125	2.403	0.017
Income	−0.016	−0.300	0.764
Duration of disease	−0.047	−0.891	0.373
Treatment type	0.003	0.059	0.953
Visit interval	−0.214	−4.054	0.000**
Participation in educational sessions	−0.084	−1.609	0.109
Source of obtaining information	0.163	3.111	0.002**
BMI	0.004	0.075	0.940
Knowledge of patients	0.100	1.711	0.088
Model 2			
Sociodemographics and the model constructs			
Visit interval	−0.176	−3.660	0.000**
Source of obtaining information	0.145	3.020	0.003**
Subjective norm	0.049	0.918	0.359
Self-efficacy	0.243	4.007	0.000**
Attitude	0.062	1.110	0.268
Intention	0.132	2.298	0.022*

*p<0.05;

**p<0.01

This study indicated the remarkable effects of the extended TRA constructs upon behavioural intention and subsequent behaviour; self-efficacy was the strongest predictor of intentions and dietary practices. These effects/relationships should be considered when designing educational programmes to promote dietary practice among diabetic patients. For instance, in order to increase type 2 diabetic patients’ motivation/intention to follow recommended diet, educational interventions first focus on changing self-efficacy of the diabetics, and then other variables of mentioned model are considered.

## ACKNOWLEDGEMENTS

The authors thank all the research assistants and Madani Hospital Diabetic Clinic staff who were involved in this study, for their support and commendable work.

## References

[1] Hussain A, Claussen B, Ramachandran A, Williams R (2007). Prevention of type 2 diabetes: a review. Diabetes Res Clin Pract.

[2] Amos AF, McCarty DJ, Zimmet P (1997). The rising global burden of diabetes and its complications: estimates and projections to the year 2010. Diabet Med.

[3] Tuomilehto J, Lindström J, Eriksson JG, Valle TT, Hämäläinen H, Ilanne-Parikka P (2001). The Finnish Diabetes Prevention Study Group. Prevention of type 2 diabetes mellitus by changes in lifestyle among subjects with impaired glucose tolerance. N Engl J Med.

[4] Knowler WC, Barrett-Connor E, Fowler SE, Hamman RF, Lachin JM, Walker EA (2002). Diabetes Prevention Program Research Group. Reduction in the incidence of type 2 diabetes with lifestyle intervention or metformin. N Engl J Med.

[5] The Diabetes Control and Complications Research Group. (1993). The effect of intensive treatment of diabetes on the development and progression of long-term complications in insulin-dependent diabetes mellitus. N Engl J Med.

[6] Monnier L, Grimaldi A, Charbonnel B, Iannascoli F, Lery T, Garofano A (2004). Mediab. Management of French patients with type 2 diabetes mellitus in medical general practice: report of the Mediab observatory. Diabetes Metab.

[7] Harris MI; National Health and Nutrition Examination Survey (NHANES III). (2001). Frequency of blood glucose monitoring in relation to glycemic control in patients with type 2 diabetes. Diabetes Care.

[8] Peyrot M, Rubin RR, Lauritzen T, Snoek FJ, Matthews DR, Skovlund SE (2005). Psychosocial problems and barriers to improved diabetes management: results of the Cross-National Diabetes Attitudes, Wishes and Needs (DAWN) study. Diabet Med.

[9] Glasgow RE, Hampson SE, Strycker LA, Ruggiero L (1997). Personal-model beliefs and social-environmental barriers related to diabetes self-management. Diabetes Care.

[10] Brug J, Oenema A, Ferreira I (2005). Theory, evidence and Intervention Mapping to improve behavior nutrition and physical activity interventions. Int J Behav Nutr Phys Act.

[11] Glanz K, Rimer BK, Viswanath K (2008). Health behavior and health education: theory, research, and practice.

[12] Bentler PM, Speckart G (1979). Models of attitude-behavior relations. Psychol Rev.

[13] Green LW, Kreuter MW (1991). Health promotion planning: an educational and environmental approach.

[14] Kulh J, Beckmann J (1985). Action control: from cognition to behavior.

[15] Bandura A (1977). Self-efficacy: toward a unifying theory of behavioral change. Psychol Rev.

[16] Giles M, Cairns E (1995). Blood donation and Ajzen's theory of planned behaviour: an examination of perceived behavioural control. Br J Soc Psychol.

[17] Ajzen I, Fishbein M (1980). Understanding attitudes and predicting social behavior.

[18] Montano DE, Kasprzyk D, Taplin SH, Glanz K, Lewis FM, Rimer BK (1997). The theory of reasoned action and the theory of planned behavior. Health behavior and health education.

[19] Ajzen I (1988). Attitudes, personality, and behavior. Chicago.

[20] Baranowski T (1992). Beliefs as motivational influences at stages in behavior change. Int Q Community Health Educ.

[21] Bandura A (1997). Self-efficacy: the exercise of control.

[22] Schwarzer R (2008). Modeling health behavior change: how to predict and modify the adoption and maintenance of health behaviors. Appl Psychol.

[23] Bandura A (2004). Health promotion by social cognitive means. Health Educ Behav.

[24] Toobert DJ, Hampson SE, Glasgow RE (2000). The summary of diabetes self-care activities measure: results from 7 studies and a revised scale. Diabetes Care.

[25] Sjoberg S, Kim K, Reicks M (2004). Applying the theory of planned behavior to fruit and vegetable consumption by older adults. J Nutr Elder.

[26] Conner M, Norman P, Bell R (2002). The theory of planned behavior and healthy eating. Health Psychol.

[27] Rovniak LS, Anderson ES, Winett RA, Stephens RS (2002). Social cognitive determinants of physical activity in young adults: a prospective structural equation analysis. Ann Behav Med.

[28] Brekke HK, Sunesson A, Axelsen M, Lenner RA (2004). Attitudes and barriers to dietary advice aimed at reducing risk of type 2 diabetes in first-degree relatives of patients with type 2 diabetes. J Hum Nutr Diet.

[29] Nejad LM, Wertheim EH, Greenwood KM (2004). Predicting dieting behavior by using, modifying, and extending the theory of planned behavior. J Appl Soc Psychol.

[30] Nguyen MN, Otis J, Potvin L (1996). Determinants of intention to adopt a low-fat diet in men 30 to 60 years old: implications for heart health promotion. Am J Health Promot.

[31] Conn VS, Tripp-Reimer T, Maas ML (2003). Older women and exercise: theory of planned behavior beliefs. Public Health Nurs.

[32] Chan YM, Molassiotis A (1999). The relationship between diabetes knowledge and compliance among Chinese with non-insulin dependent diabetes mellitus in Hong Kong. J Adv Nurs.

